# Methodological Changes in the Field of Paleogenetics

**DOI:** 10.3390/genes14010234

**Published:** 2023-01-16

**Authors:** Mikołaj Danielewski, Joanna Żuraszek, Aleksandra Zielińska, Karl-Heinz Herzig, Ryszard Słomski, Jarosław Walkowiak, Karolina Wielgus

**Affiliations:** 1 Department of Pediatric Gastroenterology and Metabolic Diseases, Poznan University of Medical Sciences, Szpitalna 27/33, 60-572 Poznan, Poland; 2 Institute of Human Genetics, Polish Academy of Sciences, Strzeszynska 32, 60-479 Poznan, Poland; 3 Research Unit of Biomedicine, Faculty of Medicine, University of Oulu, Medical Research Center, Oulu University Hospital, P.O. Box 5000, FIN-90014 Oulu, Finland

**Keywords:** ancient DNA, molecular archaeology, next generation sequencing, DNA fragmentation, DNA contamination

## Abstract

Paleogenetics has significantly changed since its inception almost forty years ago. Initially, molecular techniques available to the researchers offered minimal possibilities for ancient DNA analysis. The subsequent expansion of the scientific tool cabinet allowed for more remarkable achievements, combined has with the newfound popularity of this budding field of science. Finally, a breakthrough was made with the development of next-generation sequencing (NGS) technologies and the update of DNA isolation protocols, through which even very fragmented aDNA samples could be used to sequence whole genomes. In this paper, we review the achievements made thus far and compare the methodologies utilized in this field of science, discussing their benefits and challenges.

## 1. Introduction

Ancient DNA (aDNA) refers to genetic material recovered from ancient, partially preserved material, including parts of dead organisms, sediments, feces or microbial species. Depending on the source condition, the material extracted from aDNA can show significant levels of time- and, in most cases, environment-driven degradation. It consists mainly of ultra-short fragments, and only rare sequences longer than 200 base pairs (bp) can be found [1]. It is difficult to ascertain when exactly DNA is considered ‘ancient’ and there is no consensus on that point in the scientific community. The first ever recovered ancient DNA fragment was 140-years-old and an online dictionary proposes the age limit of 100-years since the start of the degradation processes of the sample material, i.e., from the moment of death of the organism [2,3]. Severe fragmentation and low amounts of preserved DNA are the main factors that make aDNA analysis challenging. Despite this, aDNA research has been steadily improving due to the discovery of new suitable materials, improvements in existing protocols for aDNA recovery and the development of novel tools for aDNA analysis.

Consequently, more and more challenges can be successfully tackled. aDNA research allows for comparing genomes from different periods, sometimes even from species long extinct, and for analyzing their genetic relationship with their contemporary relatives [4,5,6,7]. It opens up the possibility of observing the evolutionary processes and how they affect populations and connecting them to historical environmental changes that affected them. For example, it has been used to detect selection signals in ancient Eurasian populations and to gauge what traits were evolutionarily advantageous in human history [8]. Studying ancient genomes could also potentially lead to a better understanding of the genetic background of so-called diseases of civilization the processes involved. It also may provide insight into how variations in SNPs and more complex traits affect our health and how they impact our susceptibility to certain diseases [9,10,11].

Furthermore, tracking demographic and cultural changes in human populations from the past is possible and it is possible to ascertain the direction of migrations [12,13]. Analysis of genetic material from ancient sediments can show the composition of ancient environments and their changes. For example, analysis of sediments from permafrost in the central Yukon in Canada provided evidence that the woolly mammoth may have persisted far longer than earlier assumed [14].

Previously, approximating evolutionary distance and genetic ancestry has been limited to mapping the differences between genomes and the mitochondrial genomes of live specimens. Using aDNA analysis enables a better understanding of the origins of these changes and monitoring their spread in different populations, which allows the estimation of the migration processes that took place in the past. An excellent example of aDNA research is the investigation of the genome during domestication. Observing the emergence of discrepancies in time provides more insights than comparing domesticated species with those originating from or sharing a common ancestor. This type of research could otherwise only be conducted by analyzing the domestication process in real time, an approach that would be time-consuming and would not provide information about already domesticated species [5].

Analysis of aDNA shares some similarities with forensic genetics; in the latter case, the age of the samples is considerably shorter, while the environmental conditions may result in a similar level of damage sustained by the DNA. Therefore, the methodologies developed for one of these fields mentioned above will likely benefit the other [15]. Ultimately, however, aDNA research is limited by the availability of viable samples. Therefore, given their limited supply and non-renewable nature, it is of the utmost importance that projects involving aDNA are performed in a planned, responsible manner.

## 2. History of aDNA Research

The beginning of aDNA research can be dated to the year 1984 when short fragments of DNA were extracted and sequenced from the dried muscle of a museum specimen of the quagga—a zebra-like species considered extinct since 1883. The specimen had been preserved in salt and was 140 years old. From the many recovered fragments, most of which had a length below 500 bp, two pieces of mitochondrial DNA (117 and 112 bp in length) were sequenced [2].

Particularly impressive about this study is not only the successfully recovered aDNA for further research but also doing so without PCR, which had not yet been invented. In 1988, Svante Pääbo published a paper in which he compared the usefulness of molecular cloning and PCR in paleogenetics. In his experiment, he wrote that molecular cloning always yielded unsatisfactory concentrations of cloning products and that the obtained clones were at risk of carrying sequences from post-mortem modifications. On the other hand, PCR provided much more promising results. Two factors cause the molecular cloning disadvantage. First, they are cloning vectors that link with damaged DNA molecules (which are abundant in aged material), thus producing no clones and bacterial systems for repairing DNA that recover damaged sequences at the cost of introducing unwanted changes in base composition. 

Conversely, PCR results in much higher concentrations. Furthermore, the risk of amplifying damaged sequences is much smaller as DNA polymerases are usually slowed down or completely stopped upon encountering damage in the template strand. This leads to ‘cherry-picking’ of well-preserved aDNA molecules and their amplification [1,16].

Thanks to its high sensitivity, the implementation of PCR made aDNA sequencing substantially more straightforward, and paleogenetics entered a phase of rapid growth as it suddenly gained the interest of scientists from many different research fields. It resulted in a surge of publications, including those reporting ground-breaking achievements such as recovering fragments of aDNA millions of years old from dinosaur bones, amber inclusions and fossilized plants. However, the scientific community rejected many of these findings later as there were reasons to question their validity—some of them were proven to be a result of sample contamination, while others were impossible to replicate. Unfortunately, the high sensitivity of PCR also amplifies contaminations. It is especially true in the case of aDNA research as the concentrations of endogenic DNA are deficient, and thus, amplified contaminants can easily outnumber the desired products. Authentication of the results is even more challenging if samples are of human origin. There are plenty of possibilities for contamination, making the differentiation of endo- and exogenic material more difficult. Because of the uncharacteristically (for aDNA) long insert and the lack of independent replication of the result, it is speculated that even the recovered DNA fragment by Pääbo from an Egyptian mummy, which was previously regarded as an important pioneering achievement in paleogenetics, may have been a result of contamination [17]. To combat the growing problem of questionable authenticity, researchers proposed sets of guidelines and criteria of authenticity aimed to reduce the probability of contamination and to ensure that recovered sequences show traits characteristic of aDNA and the species they were taken from [18].

Further rapid development occurred after next-generation sequencing (NGS) techniques were introduced. Until then, researchers mainly studied single short sequences of aDNA, which, although impressive at the time, offered little helpful information. Thanks to the capabilities of NGS and the implementation of various bioinformatic tools, it soon became possible to sequence ancient genomes completely. While previously obtained information was scarce, now it was abundant. As a result, most of the workload shifted from wet laboratory work to data-to-data analysis. It also became standard practice to share all generated data globally for other research teams to use [19].

The first who used NGS in aDNA research was Poinar’s team in 2006. The team recovered DNA from a woolly mammoth mandible and sequenced 28 million bp, of which 13 million bp were identified as endogenous. The single read was, on average, 95 bp-long. While they did not sequence the whole woolly mammoth genome, they argued that it would be entirely possible considering this single specimen’s high yield of endogenous DNA. The team also analyzed the exogenic DNA and concluded that the environmental DNA was characterized by surprisingly low diversity [20].

In 2010, Rasmussen’s team sequenced the first ancient human genome from a 4000-year-old hair of a Paleo-Inuit preserved in permafrost. The team recovered 79% (2.4 billion bp) of a complete diploidal genome with an average sequencing depth of 20× and detected 351,151 high-confidence SNPs. Data analysis provided evidence for migration from Siberia to Northern America around 5500 years ago. Eighty percent of the recovered DNA was of human origin, and no contaminants of modern human DNA were detected [21].

In the same year, both Neanderthal and Denisovan genomes were sequenced. Green’s team recovered DNA from the bones from three different Neanderthal specimens from Vindija Cave in Croatia and presented a draft of a Neanderthal genome more than 4 billion bp-long. The majority of the extracted DNA was exogenous (ranging from 95 to 99%, depending on the extract). Analysis of this genome revealed evidence for a common connection of Neanderthals and anatomically modern humans. Additionally, it allowed for identification of the genomic regions that may have been subjected to positive selection of anatomically modern humans [22]. Finally, Reich and his team sequenced the genome of a previously unknown archaic hominin from a finger bone found in Denisova Cave in southern Siberia. They named these hominins ‘Denisovans’. Analysis of the sequenced genome showed that Denisovans shared a common ancestor with Neanderthals but had a different evolutionary history than Neanderthals or modern humans [23].

In 2022, genomic data from two-million-year-old sediments from Greenland were recovered, which was a new record [24]. The previous one was an over one-million-year-old sequences from two mammoth specimens reported in 2021 [25]. Table 1 shows some of the most notable achievements in ancient DNA research organized chronologically. The importance of each discovery is briefly described.

## 3. Damage of aDNA

Hydrolytic and oxidative reactions are among the most notable causes of DNA degradation. Hydrolysis can lead to DNA fragmentation in two ways. Firstly, through the destruction of phosphodiester bonds between phosphate and deoxyribose. Secondly, through cleavage of the glycosidic bond of nitrogenous bases. Deoxyribose makes this particular DNA especially susceptible to nicking due to β-elimination. The second pathway is most predominant in purines, causing DNA fragmentation [15,41,42,43,44,45,46]. In addition, the depurination rate of guanosine is higher than that of adenine [42]. In both cases, the occurrence of nicks in DNA strands close to one another leads to DNA fragmentation.

Moreover, most aDNA fragments are around ~100 bp-long since DNA wrapped around histones (146 bp per nucleosome) is less susceptible to degradation by endonucleases and thus is protected from processes occurring post-mortem in cells [15]. Additionally, hydrolysis is also the cause of the deamination of nitrogenous bases, with cytosine being the most frequently deaminated nucleotide, which turns into uracil - a chemical analogue of thymine. While this modification does not affect the integrity of DNA molecules, it alters their primary structure, thus changing the genetic readout and potentially falsifying the outcome of sequencing. Cytosine deamination occurs mainly at the single-stranded ends of DNA molecules [47].

Oxidative reactions, on the other hand, are responsible for forming atypical bases and inter-strand cross-links and cross-links between DNA and proteins. Cross-links and bases such as hydantoins block the movement of DNA polymerases along the strand serving as a template, which makes amplification and sequencing impossible [1,27,48,49]. In addition, another product of oxidation, 8-hydroxyguanine, binds complementarily to adenine instead of cytosine, which can result in transversion mutation upon the DNA molecule’s subsequent replication [49].

The rate of post-mortem changes occurring in DNA depends heavily upon environmental conditions, therefore an old, well-preserved sample (for example in permafrost) may have a similar damage profile to a recent sample from a warm and humid environment. Damage patterns show no correlations with the ages of the samples [1].

## 4. Materials, Methods and Contamination

In the beginning, little was known about the survival of DNA particles in ancient materials, and scientists often preferred soft tissues, believing that the preservation of morphological structures would ensure the preservation of genetic material [1,2,26,50]. Soft tissues are rarely preserved, as they are susceptible to microbiological degradation that renders them heavily contaminated with exogenic DNA. Hair shafts are a considerably better sample material for aDNA extraction because their primary building block, keratin, does not allow biological contaminants to pass to the inside of the tissue, which makes decontamination procedures significantly more accessible and more efficacious [51,52,53,54]. Because of this feature, the first woolly mammoth and the ancient human genomes were sequenced from hair shafts [21,30]. A clear trend emerged with the development of paleogenetics as bones and teeth became the most often used sample materials (Table 1). They are significantly more common and in a well-preserved state.

Additionally, hard tissue chemical composition may aid in preserving DNA [55,56]. One study identified the petrosal bone as the perfect sample material, showing its consistently high preservation rates of endogenous aDNA [57]. Mann (2018) suggested that while calcified dental plaque contains high amounts of genetic material from the oral microbiome, it also contains host DNA resistant to exogenous contamination [58]. With the increased knowledge of aDNA preservation, sediments from permafrost became another source of sample material. Analysis of ancient environmental DNA allows for tracking past environmental shifts and research on paleoenvironments while posing its unique challenges [14,28]. DNA recovered from ancient sediments may also provide specific information regarding individual species; for example mitochondrial DNA from sediments indicates that Denisovans may have adapted to high-attitude conditions and that they might have passed this trait onto modern humans on the Tibetan Plateau [59].

Contamination with exogenic DNA is, together with the degradation of endogenous DNA, the biggest challenge in paleogenetics. As ancient remains are most often preserved under layers of soil, they almost always contain genetic material from microorganisms from the environment. Additionally, there is always a risk of excavation site workers contaminating the material. Furthermore, contamination may occur during the transportation of samples and even in laboratory conditions. Analysis of samples from ancient human remains presents an additional difficulty as distinguishing between endogenous and exogenous human DNA is challenging with the need to authenticate the results. A similar difficulty may be encountered when analysing samples taken from museum specimens as they may be contaminated with exogenous DNA from other similarly aged exhibits. In this case, exogenous DNA can show the same characteristic damage patterns as ancient endogenous DNA, invalidating one of the commonly used authentication criteria [60].

In the past, authentication criteria relied primarily on ensuring the sterility of laboratories, performing blank controls, rejecting improbable results and independent replication of results (Table 2). However, new solutions have emerged with the development of NGS technologies and bioinformatics. Currently, bioinformatic testing tools for the post-mortem damage characteristic of aDNA (fragmentation, misincorporation of nucleotides, ratio of deaminated cytosines) and estimating heterozygosity levels for haploid chromosomes are the most important. Similarly, bioinformatic tools can also be used to estimate error rates resulting from the misincorporation of nucleotides and to ensure that retrieved sequencing data represent the genome of the sampled specimen [61,62,63].

Besides avoiding contamination with exogenous DNA, it is vital that the sample contains enough endogenous DNA to allow obtaining sequences with high enough coverage. This increases the credibility of recovered sequences and makes the estimates regarding the levels of heterozygosity more reliable which, in turn, allows to infer about the genetic diversity of past populations and, in the end, the demographic history of populations. It is important to note that coverage stems from the number of unique reads in the DNA template, thus amplification methods such as, for example, target enrichment offer limited improvements and rise clonality levels [62,64].

## 5. Extraction of Ancient DNA

**Figure 1:**
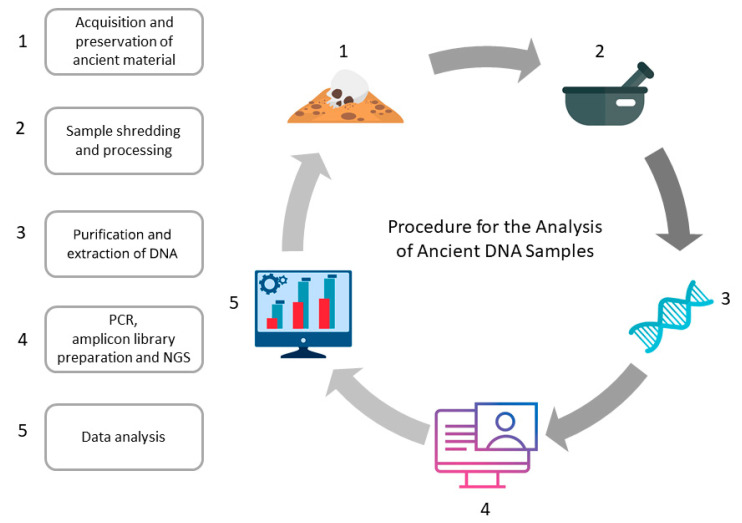
General workflow of aDNA analysis. The figure shows the order of tasks in a project regarding recovering ancient genetic material. Steps 1–4 are related to direct work with the samples either on site or in a laboratory (when potential contamination may occur); step 5 includes bioinformatic analysis, which, while represented by only a single step, is usually no less time-consuming than the other four combined. The results obtained through data analysis may indicate samples of particular interest (e.g., samples that could be re-sequenced with high coverage).

Archaeological samples of biological origin recovered from excavations may contain small amounts of endogenous DNA. However, ancient DNA is heavily degraded and modified by the impact of environmental factors, making extraction difficult and inefficient [1]. DNA extraction is a demanding process, so there are constant attempts to find the most optimal methodology. The extraction stage (Figure 1) aims to obtain sufficient DNA copies while limiting the extraction of accompanying inhibitors [65]. Different isolation techniques have been tried in the past decades, including silica bonding, ethanol/isopropanol elution, and ultrafiltration columns [66,67]. The standard isolation procedure involved powdered bone material and lysis in a buffer that disrupted cell structures [1]. According to the 1993 protocol, the lysis buffer was enriched with 10 M guanidine chloride (GuHCl), 0.1 M Tris-HCl, 0.02 M ethylenediamine tetra-acetic acid (EDTA), and Triton X-100 at a low concentration, while since 2007, there has been an optimized protocol with additional surfactants. Surfactants such as Tween, Triton X-100, sodium dodecyl sulfate (SDS), and cetyltrimethylammonium bromide (CTAB) were added interchangeably to the lysis buffer. Adding detergents did not influence the efficiency of aDNA extraction [67,68,69].

In a study involving mitochondrial DNA fragment comparative analysis, the teeth of a man who lived roughly 3000 years ago were pre-cleaned with sandpaper and sterile water. Then, the pre-treated material was pulverized in a mortar with liquid nitrogen. Lysis was performed with Tris-HCl buffer enriched with NaOH to release the surface DNA. Pioneering extractions were performed according to the standard procedure by adding guanidine thiocyanate (GTC). Unfortunately, commercial kits available at the turn of the decade did not meet the expectations for ancient DNA [70]. Two approaches to isolating ancient samples were compared, differing in the amount of bone powder used and the composition of the extraction buffer. The powdered material used during the analysis ranged from 50 to 500 mg. The first protocol proposes using a more considerable amount of powder associated with an increased DNA yield. The alkaline lysis buffer was prepared from 450 mM EDTA, 0.05% Tween, and proteinase K [66,71]. According to Dabney [72], the second protocol was optimized for bone powder equal to or less than 50 mg. The previously used Tween detergent was replaced with 1% N-lauryl sarcosine, and the volume of proteinase K was multiplied five times [72,73]. In the same year, Orlando et al. (2021)noted that pre-digestion is responsible for releasing exogenous DNA, which is mainly bound to the internal surfaces of bone [62]. The genetic material is freed from the bone by decalcification buffers that also break down organic and inorganic contaminants. Sodium phosphate or strong bleach (sodium hypochlorite) is therefore used for pre-digestion to decrease contamination with exogenous DNA. It is important to note, however, that aggressive cleaning may lead to a partial loss of endogenous DNA and thus is only recommended as an alternative [62,74].

An essential step in the extraction is the proper binding of the valuable aDNA. Silica columns and balls covered with a silica bed are the most commonly used methods of binding nucleic acids [73]. The most common type involves using silica granules combined with a supernatant. The supernatant obtained during the experimental pre-lysis is suspended in a sodium chloride binding buffer which efficiently binds the sample DNA to the silica matrix. The binding buffer was experimentally optimized by using exchangeable salts that do not affect the denaturation of the molecule [65,67,68]. Ethanol (80%) was used to wash the granules, followed by drying and elution. Dabney’s method involves purifying DNA using silica columns and a binding buffer (GuSCN, isopropanol, Tween 10 and sodium acetate). The liquid fraction is passed through QIAGEN MinElute columns, the material bound on the membrane is washed with the enclosed PE buffer, and elution from the column is washed with EB buffer [65,68].

A well-known procedure is a protocol developed by Yang et al. (1998) [75] and optimized in 2014 using Amicon Ultra (MERCK). Cellulose filters concentrate biological samples, including nucleic acids. Further purification and elution are also based on the MinElute PCR Purification Kit, designed for fragments >70 bp [68,73]. Researchers use mixed phenol-chloroform pretreatment in exceptional cases, which is complementary [62,76]. The qualitative and quantitative evaluation was typically performed by agarose gel electrophoresis and compared with modern DNA. The DNA concentration and the preparation’s purity were determined by measuring the absorption of UV light. Unfortunately, the amount of aDNA is often so small that it cannot be visualized [77]. In 2017, the results of an experiment compared the ancient DNA quantification methods. It was possible to analyze medieval bone material using absorbance, qPCR with SYBR Green detection, qPCR-commercial kit, quantitative DNA analysis using fluorescent dyes, and fragment analysis. The NanoDrop spectrophotometer and the Qubit fluorometer could simultaneously determine a high concentration of aDNA with the level of accompanying microorganisms’ DNA. Real-time polymerase chain reactions did not allow for concentration estimation by under-measurement. The most reliable method is fragment analysis, which helps to determine the concentration of DNA and the length of a given fragment [78].

## 6. Amplification

Efficient amplification requires only one copy of the target region; however, the amplification of ancient DNA is limited by the small amount of endogenous and undegraded genetic material. The polymerase chain reaction is commonly intended for modern analysis. Therefore, a high probability of duplicating additional modern accompanying microbial DNA exists. The problem can be solved partially by using special tests only for a specific species (specific PCR primers). Each cell of the fossil material may have hundreds or thousands of copies of mtDNA, which can be relatively quickly isolated and sequenced. The human mitochondrial genome has many features that make it useful in molecular analysis. It is characterized by its small size (16,569 bp), is inherited maternally and is relatively quickly evolving. The only non-coding sequence in human mtDNA is the D loop. The frequency of mtDNA mutations is 5–10 times higher than that of nuclear DNA. Therefore, it may be a source of information about the evolution of relatively recently separated species. However, mtDNA represents a single genetic locus that may not reflect the history of the entire genome [79,80]. 

DNA analysis can be performed directly by PCR or by PCR using commercially available kits after initial whole genome amplification. Whole genomic amplification (WGA) involves DNA library preparation. The final WGA product, cleaned by, e.g., CleanUp Kits, retains single- and double-stranded fragments [81]. The amplified, diverse DNA fragments can be cloned in the pGEM-T Easy vector (3018 bp) and grown in *Escherichia coli*. Selected bacterial colonies in which plasmid DNA has been detected are further selected in the presence of an optimal medium and ampicillin. After overnight incubation, the plasmid DNA and insert are isolated and sequenced.

In many cases, archival DNA analysis may involve shorter DNA fragments. In case of a known sequence for the species under study, carrying out a PCR reaction is a simple task. In absence of species-specific primers, it is possible to use amplification with primers selected for the species closest to the species of interest or choose primers for interspecies conserved regions. It is advisable to use nested PCR to visualize the result with a low amount of aDNA in some cases. Short tandem repeat (STR) analysis deals with problematic samples from ancient debris and fossils. Thanks to the method, genetic profiles of the population and racial kinship are obtained. However, one should use at least two different multiplexing kits or the same with other primer pairs. For example, for this purpose, a combination of PowerPlex^®^ ESI Promega and the NGM ™ PCR Amplification Kit (Applied Biosystems, Waltham, MA, USA) was proposed [82,83,84,85]. PCR, while initially useful in this field, has its limits, therefore it was later replaced by more advanced methods.

## 7. Target Enrichment via Hybridization-Based Capture

In the analysis of paleontological remains, capture methods are vast and used to study entire mitochondrial genomes and fragments of nuclear genomes. Capture consists of the hybridization of libraries with probes, carried out in a liquid medium-in solution or on a solid support-microarray [54]. Probes, known as baits, are single-stranded oligonucleotides specific to a given target region. According to standard experimental procedures, RNA probes are recommended for higher stability, unlike the DNA-DNA duplex. RNA baits also increase the hybridization step’s efficiency in ancient DNA research [86,87].

Hybridization on a solid substrate in work on contemporary material resulted in more than 320-fold enrichment of the DNA sequence of the exon and showed the possibility of identifying rare mutations. An analogous approach to ancient samples shows the limitations of microarray hybridization. This capture requires a relatively large amount of available aDNA [88]. The second of the previously mentioned techniques is whole-genome-in-solution capture (WISC) to propagate the increase of endogenous aDNA in amplicon libraries. The capture allows samples to be enriched 50-fold, with initially low concentrations of human genetic material [89]. The purpose of the WISC method is based on the use of specific biotinylated baits in constructing libraries by transcribing fragmented genomic DNA [90]. Biotinylated probes are targeted at the control regions of mitochondrial DNA, a genetic marker in studying ancient material due to many copies. [91,92].

The general capture mechanism involves binding bait to a target sequence that captures a DNA fragment from the solution while maintaining appropriate characteristics such as particle size and damage pattern. Capturing the original damage pattern is extremely important in paleontological research to determine the correct age [93]. In 2017, WISC coupled with shotgun sequencing for DNA analysis of extinct species of a wild and domestic dromedary was used. The analysis showed a significant increase in the percentage of mtDNA in the samples, which was directly related to the 187-fold increase in mapped reads. In addition, a complete mitochondrial genome image was obtained for one example. The increase also contributed to the recovery of endogenous nuclear DNA [94].

Targeting worked particularly well in paleomicrobiology, which holds information on the genetic evolution of bacteria and viruses or interacts with the human population.In the last decade, a breakthrough was achieved. The entire pPC1 plasmid sequence and most genomes of *Yersinia pestis*, the virulence factor responsible for the outbreak of the plague pandemic in the 6th and 14th centuries, were recreated [32,95,96]. This success was repeated with the genome of *Y. pestis* from the late Bronze Age in today’s Russia. It provided new information on the transmissibility of plague by fleas [97].

Target enrichment through hybridization-based capture is a powerful tool for reconstructing the genome of humans, animals, plants, microorganisms, or entire ecosystems. This technology helps to obtain information on origin, genetic diversity, evolutionary features, environment, epidemiology, or host-pathogen interaction. Currently, Daicel Arbor Biosciences’ myBaits^®^ off-the-shelf target capture kits are commercially available, providing a package of biotinylated probes in solution and reagents for highly efficient and targeted sequencing on any platform, namely Illumina, PacBio, and Nanopores [86]. However, commonly available procedures require constant optimization regarding variables such as purification and sample fragmentation, the number of amplification cycles, the hybridization time, and the library size. In addition, the process itself is often time-consuming due to the long time it takes to create probes from reference genomes [98].

## 8. Sequencing

Capillary sequencing was the standard genotyping method in the early days of aDNA. Sanger sequencing worked well for small fragment analysis but had limitations due to low throughput and high costs [99]. Hence, the emerging problems and methodological limitations in genomic research required the introduction of new sequencing methods. In 2006, next-generation sequencing technology (NGS) appeared on the biotechnology market [100]. With the NGS method, billions of readings of the DNA sequence can be obtained. The entire genome of the tested individual can be sequenced at a lower unit cost. An essential advantage of NGS is the easy data extraction from short aDNA fragments (30–100 bp) despite the often-problematic PCR amplification.

The basis of the procedure is the construction of amplicon libraries, which may be limited by accompanying PCR inhibitors, damaged nitrogen bases, and the undoubtedly low copy number of endogenous aDNA. Such limitations directly affect the conversion efficiency of DNA fragments [101]. Regarding library preparation, two methodological approaches can be distinguished: A double-stranded library and a single-stranded library. The double-stranded amplicon library developed initially is prone to incomplete ligation and damage, while its construction requires the ligation of double-stranded adapters to aDNA fragments with repaired ends [102,103]. The Illumina Company introduced modifications where each aDNA molecule was ligated to separate adapters with no significant loss of genetic material [104]. Gansauge and Meyer, (2013) also developed a single-stranded library dedicated to ancient material [102]. The single-stranded molecule ligation with a biotinylated adapter at the 3’ end using CircLigase and the binding of the product on streptavidin-coated beads were described in 2013. The use of such beads avoids DNA loss during the purification steps. In the same year, the enzyme was replaced with T4 DNA ligase, and an oligonucleotide with a random base sequence with a biotin-labelled adapter was introduced. Despite the many advantages of the single-stranded library strategy, it is time-consuming and expensive [102,105]. Research on ancient biological samples has shown that the benefits of choosing a single-stranded protocol are particularly apparent when working with problematic/contaminated samples. Thanks to the continuous optimization and modifications of the described strategies, it is possible to sequence the genome more efficiently and thus obtain information about ancient species [106,107].

## 9. Where Paleogenetic and Forensic Sciences Converge

Since DNA degradation is dependent on many factors in addition to time, genetic material from samples studied in forensic science can be as heavily degraded as the one of ancient origin. Thus, the methodology used in paleogenetics could be used in forensic science. However, as severe degradation of DNA is less of a consistent problem in forensic genetics than in ancient genetics, the focus has been on the improvement of short tandem repeat polymorphisms that require the amplification of fragments up to almost 300 bp-long. In addition, new forensic methods must undergo a rigorous testing phase before being implemented, which has not been the case for ancient DNA methods. However, the feasibility of introducing ancient DNA methods into forensic methodology has recently been published. Authors have shown that implementing such methods improves forensic DNA profiling and they argue that it can increase the success rate for identifying historical remains [56,108].

Another problem that forensic and ancient genetics share is the necessity of damaging the remains to obtain the required samples. As human remains pose high emotional and historical value, it is of utmost importance to limit the damage to a bare minimum. In 2016, a method that allows avoiding damaging a bone sample’s surface was described. The method entails drilling a tiny hole through the bone and inserting two needles in the resulting openings. The needles are fixed to the bone with polyester resin, and the bone itself is covered in aquarium sealant to prevent the eluent from escaping through possible pores (both polyester resin and the sealant can be easily removed from the bone without further damage). An infusion pump delivers elution fluid at 56 °C via a thinner needle into the bone. The outlet needle should be thicker to prevent hydrostatic pressure from rising inside the bone. While this method yields only 5% of the amount of DNA that the destructive method would yield, it should be noted that this amount still allows DNA profiling. It had been successfully utilized to isolate DNA from skulls from a mass grave to identify the remains [109].

## 10. Conclusions

The field of paleogenetics has undergone some drastic changes. While initially limited to analyses of singular short DNA sequences, it is now possible to sequence whole genomes and perform metagenomic studies. Despite the initial problems with the authentication of the findings, paleogenetics has become a new field. Among the essential improvements in the methodology were incorporating NGS technologies, developing protocols for isolating highly fragmented DNA, and establishing standards for verifying results. With further development of NGS technologies and their increasing affordability, the scientific community will continue to analyze ancient genomic data to achieve the finest possible resolution of the genetic history of our world. Additionally, as genomic data alone hardly offer a complete view, scientists in the future will most likely focus on multi-field studies based on paleogenetics, paleo-proteomics, metagenomics, and possibly paleo-epigenetics.

## Figures and Tables

**Table 1:** Notable achievements in the field of ancient DNA research.

Year of Discovery	Methods	Sample Material	Result	References
1984	Molecular cloning, Sanger’s sequencing	Dried muscle tissue, quagga specimen	Two sequenced mitochondrial DNA fragments (117 and 112 bp). First recovered aDNA.	[2]
1988	PCR	Dried muscle, quagga specimen	Detected cloning artefacts previously unnoticed in [2] with PCR.	[16]
1988	Molecular cloning, PCR	Numerous different ancient samples	Comparing the usefulness of molecular cloning and PCR in aDNA research.	[1]
1991	PCR	Human brain tissue, 6990–8130 years old	Sequenced fragments of 6 nuclear genes.	[26]
1998	PCR	Coprolite	Amplification of DNA from ancient feces. Analysis of the diet of the specimen and identification of species of the specimen.	[27]
2003	PCR	Sediment	First analysis of environmental aDNA	[28]
2005	PCR	Bones, teeth	Intact stretches of mitochondrial DNA from 24 Neolithic skeletons.	[29]
2006	NGS	Woolly mammoth’s mandible	28 million bp sequenced, 13 million bp were endogenous. First use of NGS in paleogenetics. Analyses of the metagenomic nature of ancient remains.	[20]
2008	NGS	Woolly mammoth’s hair	4.17 billion bp sequenced, 3.3 billion of which were endogenous	[30]
2008	NGS	Neanderthal bone	Fully sequenced Neanderthal mitochondrial genome	[31]
2010	NGS	21 Neanderthal bones, 3 selected for further analysis	First sequenced Neanderthal genome (1.2× coverage), evidence for Neanderthals interbreeding with anatomically modern humans	[22]
2010	NGS	Finger bone	Discovery of Denisovans and sequenced Denisovan genome	[23]
2010	NGS	Hair	First sequenced ancient human genome (Paleo-Inuit)	[21]
2011	NGS	Teeth, bones	First fully sequenced genome of ancient bacterial pathogen	[32]
2012	NGS	Finger bone	First high coverage (30×) of Denisovan genome, use of single-stranded library preparation.	[33]
2012	NGS	Bone from the mummy of Tyrolean Iceman	Genome of Tyrolean Iceman fully sequenced, analysis of phenotype and metagenome	[34]
2014	NGS	Ancient calcified dental plaque	First high-resolution taxonomic and proteomic analysis of ancient oral microbiome from calcified dental plaque	[35]
2014	NGS	Bones	Identification of English king Richard III	[36]
2014	NGS	Toe phalanx	High-quality sequence of Neanderthal woman genome (coverage ~50×)	[37]
2015	NGS	-	Analysis of 230 ancient Eurasian genomes to determine genome-wide patterns of selection	[8]
2015	NGS	Molar tooth, soft tissue	Complete high-quality two woolly mammoth genomes, analysis of demographic history	[6]
2015	NGS	Auroch bone	6750-year-old auroch genome, analysis of domestication process and its impact on the genome	[5]
2017	NGS	Bone	High-coverage genome (30×) of Neanderthal from Vindija Cave, analysis of gene flow between Neanderthals, Denisovans and anatomically modern humans	[38]
2020	NGS	-	Sequencing of 442 genomes from archaeological sites across Europe and Greenland to understand the expansion of the Scandinavian population during the Viking Age	[39]
2020	NGS	Finger bone	High coverage (27×) sequencing of a Neanderthal from Chagyrskaya Cave. Detection of selection patterns in Neanderthal lineage	[40]
2021	NGS	Loessal permafrost silts	Analysis of ancient sedimentary DNA from a period of 30,000 years from the central Yukon in Canada.	[14]
2021	NGS	Mammoth molars	Previous record for the oldest sequenced genome (older than 1 million years).	[25]
2022	NGS	Sediment	Current record holder for the oldest sequenced DNA	[24]

**Table 2:** Authenticity criteria. The table shows the criteria to follow to ensure that the recovered sequences are indeed ancient and not a result of contamination with modern genetic material [61,63].

Criterion	Explanation
Physically isolated work area	All work on low-copy number DNA should be carried out in an isolated laboratory where no other genetic research is performed.
PCR control amplifications	Test laboratory environment for contamination.
Test the molecular behavior	Check the PCR products for unusual results. aDNA is heavily fragmented, so longer fragments should be increasingly rarer.
Quantitation	Check the number of starting templates. If below 1000, sporadic contamination cannot be ruled out.
Reproducibility	Results from the same sample material should be repeatable.
Clone	After sequencing, the PCR product should be cloned and sequenced in multiple copies to determine the ratio of exogenous sequences and sequencing errors resulting from aDNA damage.
Independent replication	The results should be reproduced in another independent laboratory.
Biochemical preservation	Survival of other ancient biomolecules makes the survival of aDNA more believable.
Associated remains	If target DNA sequences also survive in associated faunal material, it may be used as supporting evidence.
Phylogenetic sense	Reproducible sequences should be placed in a phylogenetic tree with other known haplotypes.
Damage patterns	The DNA sequences should show specific damage patterns: a high degree of fragmentation and a high concentration of substitutions on the ends of the fragments (C>T on 5′ and G>A on 3′).

## Data Availability

No new data were created or analyzed in this study. Data sharing is not applicable to this article.
